# On Connectivity of Wireless Sensor Networks with Directional Antennas

**DOI:** 10.3390/s17010134

**Published:** 2017-01-12

**Authors:** Qiu Wang, Hong-Ning Dai, Zibin Zheng, Muhammad Imran, Athanasios V. Vasilakos

**Affiliations:** 1 Faculty of Information Technology, Macau University of Science and Technology, Macau, China; qiu_wang@foxmail.com; 2 School of Data and Computer Science, Sun Yat-Sen University, Guangzhou 510006, China; zhzibin@mail.sysu.edu.cn; 3 College of Computer and Information Sciences, King Saud University, Riyadh 12372, Saudi Arabia; dr.m.imran@ieee.org; 4 Department of Computer Science, Electrical and Space Engineering, Lulea University of Technology, Luleå 971 87, Sweden; vasilako@ath.forthnet.gr

**Keywords:** connectivity, wireless sensor networks, directional antennas

## Abstract

In this paper, we investigate the network connectivity of wireless sensor networks with directional antennas. In particular, we establish a general framework to analyze the network connectivity while considering various antenna models and the channel randomness. Since existing directional antenna models have their pros and cons in the accuracy of reflecting realistic antennas and the computational complexity, we propose a new analytical directional antenna model called the iris model to balance the accuracy against the complexity. We conduct extensive simulations to evaluate the analytical framework. Our results show that our proposed analytical model on the network connectivity is accurate, and our iris antenna model can provide a better approximation to realistic directional antennas than other existing antenna models.

## 1. Introduction

Wireless sensor networks (WSNs) have often been deployed in many areas without infrastructure support. The nodes in WSNs communicate with each other in an ad hoc manner. When the destination is far from the source, data packets will be relayed through multiple intermediate nodes in a multi-hop fashion. Compared with infrastructure wireless networks, such as Wireless Local Area Networks (WLANs) and cellular networks, WSNs are more susceptible to the failure of nodes due to various reasons, such as channel fading, the depletion of the battery power and malicious attacks. As one of the most important metrics to evaluate the reliability of WSNs, the network connectivity concerns the possibility that a node can establish a successful communication with another node, which is essentially the prerequisite for designing the effective topology control schemes in WSNs.

Many previous studies investigated the network connectivity of WSNs under the assumption that each node is equipped with omni-directional antennas. However, omni-directional antennas radiate/receive radio signals in all directions [1,2]. As a result, WSNs equipped with omni-directional antennas suffer from poor network performance due to the interference on some undesired directions and the short transmission range.

Recent works, such as [3,4,5,6,7,8], show that applying directional antennas in WSNs can greatly improve the network performance. The performance improvement mainly is owed to the effect that directional antennas can concentrate the radio signal in the desired directions so that the interference in other undesired directions is significantly reduced. As a result, directional antennas have become mandatory in the future generation communication systems, such as millimeter-wave (mmWave) networks [9,10,11,12,13] and 802.11ad WiFi [14,15] in order to compensate the high attenuation of mmWave signals. Besides, directional antennas can be deployed in robotic sensor networks to achieve a longer communication range and lower interference than omni-directional antennas [16,17]. Furthermore, it is shown in [7,18,19] that WSNs with directional antennas have higher network connectivity than WSNs with omni-directional antennas. However, the studies in [18,19] only consider the realistic antenna models, which are so complicated that they are neither appropriate for analytical study, such as obtaining the optimal bounds of the network connectivity in [20,21], nor applicable to the design of directional MAC protocols [22,23,24,25]. To address these issues, two simplified analytical directional antenna models—the sector model [22,26] and the keyhole model [20,27,28]—were proposed. However, both the sector model and keyhole model are somewhat over-simplified and therefore fail to capture the important features of directional antennas. For example, the keyhole model cannot depict the nulling capability of realistic antennas, while the sector model ignores the side/back lobes of realistic antennas.

In this paper, we propose a novel analytical directional antenna model, which can approximate realistic antennas while maintaining a certain simplicity. We name this model the iris model since it is geometrically analogous to an iris flower, where the main beams of an antenna are analogous to the petals of the iris flower and the side/back lobes are analogous to the sepals of the flower.

Our proposed iris model has many merits over those existing simplified antenna models, including the keyhole and sector models. Table 1 summarizes these benefits of our proposed iris model in contrast to other existing models. In particular, similar to the keyhole model, the iris model can approximate both the main beam and side/back lobes of realistic antennas, while the sector model cannot. Besides, the iris model can depict the nulling capability of realistic antennas, albeit the keyhole model cannot. Note that the sector model overestimates the nulling capability. Moreover, the iris model can approximate directional antennas with more than one main beam, while both the keyhole model and sector model cannot. Last, but not least, the iris model is simpler and tractable in contrast to realistic antenna models.

In this paper, we conduct a comprehensive study on the network connectivity with the consideration of various antenna models. The main research contributions of this paper can be summarized as follows.

1. We establish a general framework to analyze the network connectivity with various existing directional antenna models and our proposed iris model. In particular, we investigate both the local connectivity and the overall connectivity of WSNs in the presence of channel randomness. More specifically, the local connectivity mainly concerns the probability of the node isolation of a node, while the overall connectivity evaluates the probability that there exists at least one path for each node pair in the network from the viewpoint of the entire network.
2. We conduct extensive simulations to validate the analytical framework and evaluate the accuracy of the existing antenna models and our proposed model. Our simulation results match the analytical results, indicating that the analytical framework is quite accurate and effective. Besides, our proposed iris model provides a relatively better approximation to realistic antennas than the keyhole model and the sector model on average.
3. We find that the network connectivity heavily depends on different antenna models and different channel conditions. We demonstrate that the channel randomness (such as the path loss and the shadow fading) has significant impacts on the network connectivity. For example, the path loss effect is always detrimental to the network connectivity, and the shadow fading effect is somewhat beneficial to the connectivity.

The rest of this paper is organized as follows. We first present a survey on related works in Section 2. Section 3 then introduces the antenna models used in this paper, and Section 4 gives the channel models. We next present both the analytical results and the simulations results on the local network connectivity in Section 5. Section 6 analyzes the overall network connectivity. We discuss the applications of our proposed models and the future directions in Section 7. Section 8 concludes this paper.

## 2. Related Works

The network connectivity of WSNs has received considerable research attention recently. In particular, there are a number of studies on the network connectivity of WSNs. More specifically, reference [29] investigated the problem of deriving a sufficient and necessary condition to ensure that the network is connected. Bettstetter analyzed the network connectivity with probability models in [2]. The work in [30] is the extension of [2] with the consideration of shadow fading effects. Besides, the impacts of various random channel models on the network connectivity of WSNs were considered in [31]. A survey on the coverage and connectivity issues in WSNs was presented in [32]. The local connectivity of wireless cognitive radio networks was investigated in [33]. However, most of these studies only consider the WSNs with omni-directional antennas, which radiate/receive signals in all directions, including some undesired directions, and consequently result in the poor network performance.

Using directional antennas instead of omni-directional antennas in wireless ad hoc networks or WSNs can significantly improve the network performance since directional antennas can concentrate the transmitting/receiving capability to desired directions [34]. For example, it is shown in some recent studies that using directional antennas in WANs can improve the network capacity and reduce the end-to-end delay [3,4,5,6,7,8,26]. Besides, using directional antennas in WSNs can only improve the security, as shown in [35,36]. In WSNs with directional antennas, it is difficult for each node to obtain the location knowledge of other neighbors due to the directional beamforming [34]. To solve the problem of directional neighbor discovery, the complicated schemes, such as using direction-of-arrival estimation, swiveling the beam from 0 to 2π, were proposed [37]. Random beamforming schemes have relatively lower communication overhead than the neighbor-discovering schemes. Specifically, the empirical study on the network connectivity with random beamforming scheme was first conducted in [38]. A lightweight scheme named the center-directed beamforming scheme was proposed in [39]. A comparison study with various beamforming schemes and channel randomness was conducted in [19]. The work in [40] investigated the asymptotic connectivity of WSNs with directional antennas. Moreover, reference [41] investigated the connectivity of wireless networks, in which multiple directional antennas are equipped with each node.

One of important issues with directional antennas lies in the directional antenna models. In particular, realistic directional antenna models were used in previous works, such as [19,42]. However, these models are so complicated that they are not tractable in the performance analysis. For example, it is shown in [20] that it is difficult to derive the optimal bounds of the network connectivity based on realistic antenna models. To tackle this problem, several simplified antenna models, such as the sector model and keyhole model, were proposed to approximate realistic directional antennas [34]. However, the above antenna models are over-simplified. For example, the side/back-lobes have not been considered in the sector model, while keyhole ignores the nulling capability. Therefore, the objective of this paper is to propose a novel directional antenna model to approximate realistic antenna models while maintaining the key features of directional antennas.

In our previous paper [43], we conducted a preliminary study on the network connectivity in terms of the path probability. However, this paper is significantly different from our previous work [43] in the following aspects: (i) we investigate the network connectivity within a general theoretical framework, in which we consider the channel randomness and various directional antenna models; (ii) we analyze both the local connectivity (in Section 5) and the overall connectivity (in Section 6); (iii) we conduct extensive simulations to verify the effectiveness and the accuracy of our analytical model with consideration of both the local connectivity and the overall connectivity.

## 3. Antenna Models

In this section, we describe the antenna models that will be used throughout this paper. In order to model the directivity of antennas, we introduce the definition of antenna gain. The antenna gain of an antenna can be expressed in a spherical coordinate system as follows [44].

(1) $$G\left(\theta ,\phi \right)=\eta \frac{U(\theta ,\phi )}{U_{o}},$$

where *η* is the efficiency factor, which is set to be one since antennas are often assumed to be lossless, *θ* is the elevation angle from the *z*-axis within [0,π], *ϕ* is the azimuth angle from the *x*-axis in the xy-plane within [0,2π], U(θ,ϕ) is the radiation intensity, which is defined as the power radiated from an antenna per unit solid angle, and $U_{o}$ is the radiation intensity of an omni-directional antenna with the same radiation power.

### 3.1. Isotropic Antenna

We use an isotropic antenna to model an omni-directional antenna, in which it radiates/receives the radio signals uniformly in all directions in 3D space. It is obvious that the antenna gain of the isotropic antenna, denoted by $G_{o}$, is $G_{o}=1$ since $U\left(\theta ,\phi \right)=U_{o}$ in Equation (1).

### 3.2. Directional Antennas

A directional antenna radiation pattern typically consists of main lobes with the largest radiation intensity and side or back lobes with smaller radiation intensity. To accurately depict a directional antenna, we introduce the following properties:

- The radiation beam (lobe) is a clear peak in the radiation intensity surrounded by regions of weaker radiation intensity.
- The Half Power Beam Width (HPBW) is the angular width between the half-power (−3 dB) points of the lobe.
- The main beam represents the radiation lobe with the maximum antenna gain.
- The side or back lobes represent the lobes in any directions other than the direction of the main beam.
- The nulling capability is the capability of a directional antenna employing nulls to counteract unwanted interference in some undesired directions.

**Figure 1:**
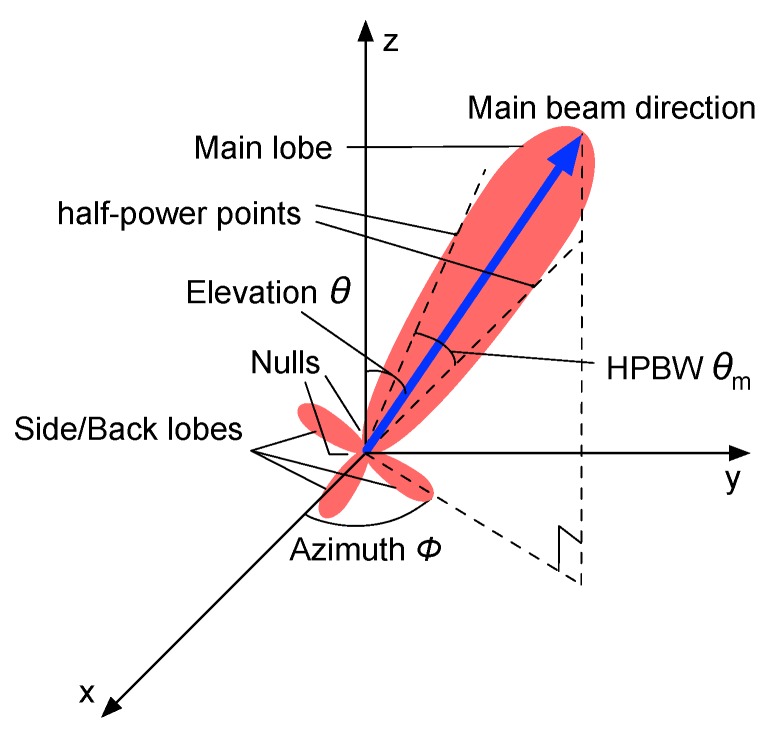
Realistic directional antenna.

Figure 1 shows an example of a directional antenna radiation patter, in which there is one main lobe, several side or back lobes and several nulls.

To calculate the antenna gain of an antenna, we introduce the radiation power $P_{rad}$, which is defined in [44],

(2) $$P_{rad}=\underset{\Omega }{\int \;\;\;\;\;\int \;◯}U\left(\theta ,\phi \right)d\Omega =\int_{0}^{2\pi }\int_{0}^{\pi }U\left(\theta ,\phi \right)\sin\theta d\theta d\phi ,$$

where Ω is the steradian, *θ* is the elevation angle and *ϕ* is the azimuth angle.

It is obvious that an isotropic antenna has a constant radiation intensity $U_{o}$ since it radiates power in all directions. We then have $P_{rad}=4\pi U_{o}$ by the integration with Equation (2), which implies $U_{o}=\frac{1}{4\pi }P_{rad}$. After replacing $U_{o}$ in Equation (1) by $\frac{1}{4\pi }P_{rad}$ and replacing $P_{rad}$ by the integration of Equation (2), we then express Equation (1) as follows,

(3) $$G\left(\theta ,\phi \right)=\frac{U(\theta ,\phi )}{\frac{1}{4\pi }\int_{0}^{2\pi }\int_{0}^{\pi }U\left(\theta ,\phi \right)\sin\theta d\theta d\phi }.$$

In this paper, we consider two kinds of typical directional antennas: Uniform Circular Array (UCA) antennas [37,42,45,46] and Uniform Linear Array (ULA) antennas [46,47], which are introduced in detail as follows.

#### 3.2.1. Uniform Circular Array

**Figure 2:**
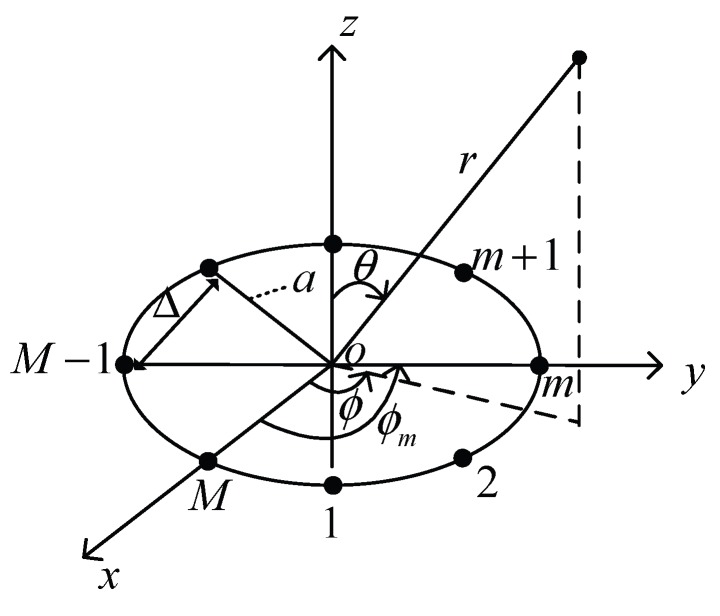
Structure of a Uniform Circular Array (UCA) antenna.

Figure 2 shows an example of a UCA antenna, in which there are *M* isotropic antenna elements equally spaced on the xy-plane along a circle of radius *a*. In this structure, any two neighboring elements are separated with a distance Δ ranging from λ/2 to *λ* (where *λ* is the wavelength of signal). The radiation intensity of a UCA antenna fulfills the following formula [44]:

(4) $$U\left(\theta ,\phi \right)\propto {\left|E\left(\theta ,\phi \right)\right|}^{2},$$

where E(θ,ϕ) is the far-zone electric-field strength of the antenna at a given direction (θ,ϕ), which is expressed as:

(5) $$E\left(\theta ,\phi \right)=\sum_{m=1}^{M}I_{m}e^{jka[\sin\theta \cos\left(\phi -\phi_{m}\right)-\sin\theta_{0}\cos\left(\phi_{0}-\phi_{m}\right)]},$$

where *j* is the imaginary unit, k=2π/λ, $\phi_{0}$ ($\phi_{0}\in \left[0,2\pi \right]$) and $\theta_{0}$ are respectively the azimuth angle and elevation angle, $\phi_{m}=2\pi m/M$ is the angular position of the *m*-th element on the xy-plane and $I_{m}$ is the amplitude excitation of the *m*-th element, which is set to be one [19].

After replacing U(θ,ϕ) in Equation (3) by Equation (4), the antenna gain of UCA antennas can be expressed as follows,

(6) $$G\left(\theta ,\phi \right)=\frac{{\left|E\left(\theta ,\phi \right)\right|}^{2}}{\frac{1}{4\pi }\int_{0}^{2\pi }\int_{0}^{\pi }{\left|E\left(\theta ,\phi \right)\right|}^{2}\sin\theta d\theta d\phi }.$$

**Figure 3:**
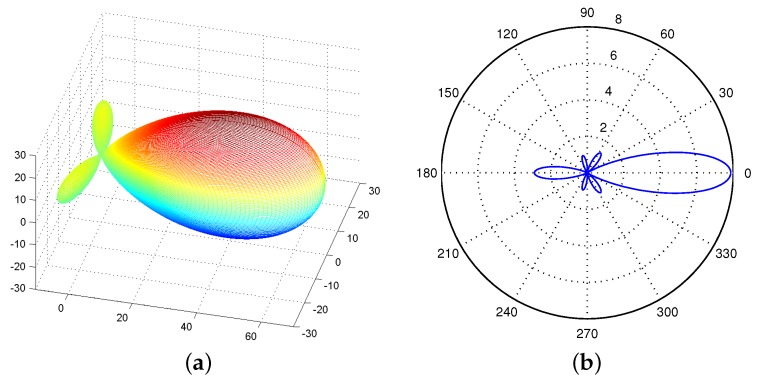
Radiation pattern of a UCA antenna. (**a**) In 3D space; (**b**) on a 2D plane.

Figure 3a shows the radiation pattern of the UCA antenna in 3D space based on the above derivation. Since we are concerned with the network connectivity of WSNs in the 2D plane, the antenna gain of UCA antenna in 3D space is projected to the xy-plane by setting θ=π/2 and $\theta_{0}=\pi /2$. Figure 3b shows the projection.

#### 3.2.2. Uniform Linear Array

**Figure 4:**
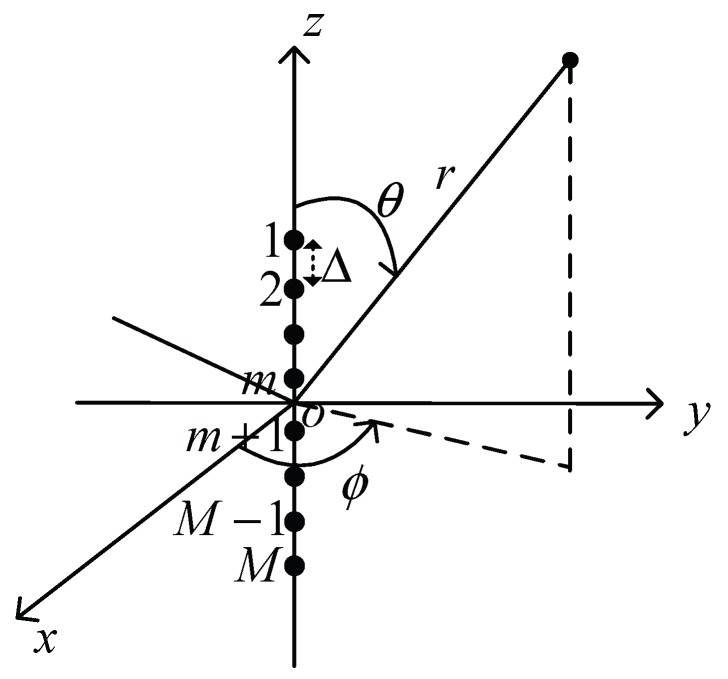
Structure of Uniform Linear Array (ULA) antenna.

Figure 4 shows the structure of a ULA antenna that consists with *M* isotropic elements equally placed along a line. In this structure, any two neighboring elements are also separated by the distance Δ. The radiation intensity of a ULA antenna fulfills the following formula [38],

(7) $$U\left(\theta ,\phi \right)\propto {\left(\frac{1}{M}\frac{\sin(M\psi )}{\sin(\psi )}\right)}^{2},$$

where *ψ* is given by:

(8) $$\psi =\frac{\pi \Delta }{\lambda }\left(\cos\theta -\cos\theta_{0}\right),$$

where *λ* denotes the wavelength of signal radiated from the antenna elements, Δ is usually chosen as λ/2 and $\theta_{0}$ is the azimuth angle of the desired main beam. Note that due to the rotational symmetry structure of the ULA antenna (as shown in Figure 4), the antenna gain is independent of *ϕ*.

Thus, the gain of a ULA antenna is expressed as:

(9) $$G\left(\theta \right)=\frac{{\left(\frac{1}{M}\frac{\sin(M\psi )}{\sin(\psi )}\right)}^{2}}{\frac{1}{2}\int_{0}^{\pi }{\left(\frac{1}{M}\frac{\sin(M\psi )}{\sin(\psi )}\right)}^{2}\sin\theta d\theta }.$$

**Figure 5:**
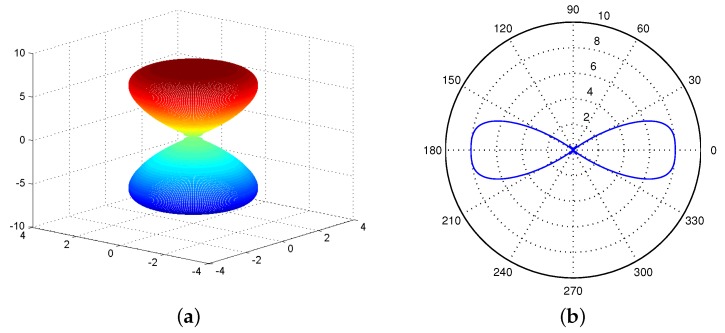
Radiation pattern of a ULA antenna. (**a**) In 3D space; (**b**) on a 2D plane.

Figure 5a shows the radiation pattern of a ULA antenna in 3D space with $\theta_{0}=0$. Similar to the UCA antennas, we also project the radiation pattern in 3D space to a xy plane and then obtain a 2D radiation pattern of a ULA antenna. Figure 5b shows an example of the 2D radiation pattern of a ULA antenna. Differently, a ULA antenna consists two main lobes while a UCA antenna has one main lobe.

### 3.3. Existing Simplified Models of Directional Antennas

The aforementioned realistic directional antenna models, such as the UCA antenna and ULA antenna, are so complicated that they may not be tractable in some studies [22,27]. Thus, several simplified directional antenna models have been proposed to address this issue. There are two typical models of simplified directional antenna listed as follows.

**Figure 6:**
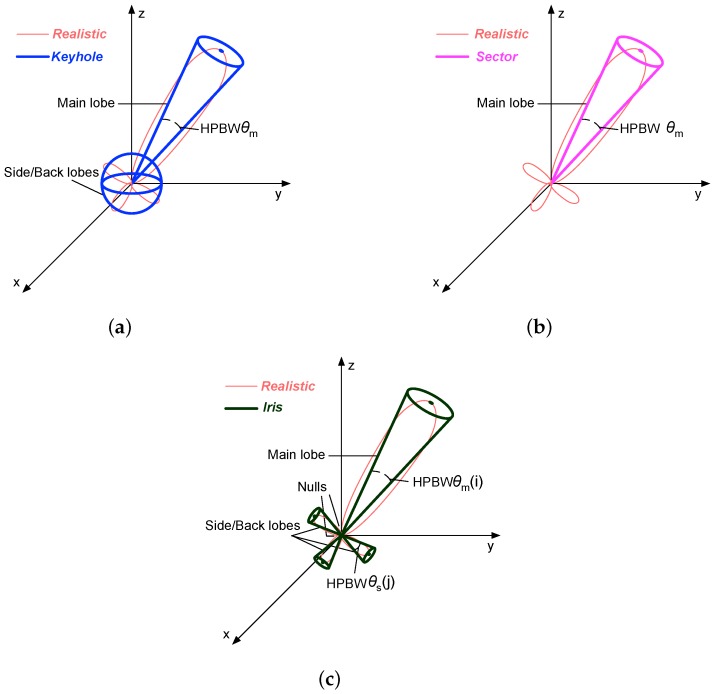
Directional antenna models. (**a**) Realistic vs. keyhole; (**b**) Realistic vs. sector; (**c**) Realistic vs. iris.

- The keyhole model consists of one main beam and multiple side/back lobes in other directions, as shown in Figure 6a. This model has been used in [11,12,27].
- The sector model consists of only one main beam and has no side/back lobes, as shown in Figure 6b. This model has been used in [22,26].

We then briefly describe the keyhole model. In particular, the radiation power $P_{rad}$ consists of two parts: (1) the main lobe part denoted by $P_{m}$; and (2) the side/back lobe part denoted by $P_{s}$. Thus, we have,

(10) $$P_{rad}=P_{s}+P_{m},$$

where $P_{rad}=4\pi U_{0}$.

The value of $P_{m}$ can be calculated by the following integral equation,

(11) $$P_{m}=\int_{0}^{2\pi }\int_{0}^{\frac{\theta_{m}}{2}}G_{m}U_{0}\sin\theta d\theta d\phi ,$$

where $G_{m}$ is the gain of the main lobe.

The value of $P_{s}$ can be calculated by the following integral equation,

(12) $$P_{s}=\int_{0}^{2\pi }\int_{\frac{\theta_{m}}{2}}^{2\pi }G_{s}U_{0}\sin\theta d\theta d\phi ,$$

where $G_{s}$ is the gain of side lobes.

Combining Equations (10)–(12) together, we can have:

(13) $$G_{s}=\frac{2-G_{m}\left(1-\cos\left(\frac{\theta_{m}}{2}\right)\right)}{1+\cos\frac{\theta_{m}}{2}}.$$

As shown in Equation (13), $G_{s}$ is a function of the antenna gain of the main lobe $G_{m}$ and the beamwidth $\theta_{m}$. In particular, when $G_{s}=0$, the keyhole model becomes the sector model.

Although both keyhole and sector models can simplify the representation of antenna radiation patterns of realistic directional antennas, they are too coarse to accurately depict a realistic antenna. Specifically, the keyhole model cannot depict the nulling capability of a realistic antenna, which nevertheless is an important feature to effectively reduce the interference. The sector model ignores the side/back lobes and overestimates the nulling capability of a realistic antenna. Besides, either the keyhole model or the sector model can only be used to approximate an antenna with a single main beam, such as UCA antennas. They cannot be applied to the ULA antenna, which often consists of two main lobes. Therefore, we next propose an antenna model, which is simple and abstract, but still reflects the main characteristics of a realistic antenna.

### 3.4. Iris Antenna Model

To overcome the limitations of existing antenna models, such as keyhole and sector models, we propose a new directional antenna model to approximate the radiation pattern of realistic antennas. We name this model the iris model since it is geometrically analogous to an iris flower. Figure 6c shows our iris model, in which the sectoral main beams are analogous to the petals of an iris flower, and the side/back lobes are analogous to the sepals of the flower. We then formally define the iris model as follows.

**Definition** **1.** *The iris antenna model consists of main beams with gains* $G_{m}\left(i\right)$*, several side/back lobes with gain* $G_{s}\left(j\right)$ *and nulls with zero gain. Specifically, the antenna gain* G(θ) *at a specific direction can be calculated by:*

(14) $$\begin{array}{c} G\left(\theta \right)=\left\{\begin{array}{cc} G_{m}\left(i\right) & within\;HPBW\;\theta_{m}\left(i\right)of\;each\;main\;lobe\\ G_{s}\left(j\right) & within\;HPBW\;\theta_{s}\left(j\right)of\;each\;side/back\;lobe\\ 0 & otherwise. \end{array}\right. \end{array}$$

Note that the gain $G_{m}\left(i\right)$ can be obtained by realistic antennas through Equation (6), though it is much simpler than realistic antennas, since it is a constant within $\theta_{m}$. Similarly, the gain $G_{s}\left(j\right)$ of each side/back lobe can also be obtained by realistic antennas through Equation (6). However, each $G_{s}\left(j\right)$ is not necessarily identical. Besides, HPBW $\theta_{s}\left(j\right)$ of each side/back lobe is also not necessarily identical. Essentially, HPBW $\theta_{s}\left(j\right)$ only depends on the corresponding HPBW of each lobe of realistic antennas. Moreover, there may exist more than one main beam for a directional antenna. Take Figure 5b as an example again, in which a ULA antenna has two main beams. One of the merits of our iris model lies in the generality since it can depict both the antennas with a single main beam and the antennas with multiple main beams while existing simplified models, such as keyhole and sector models, cannot. For presentational simplicity, we name our approximated model of a UCA antenna as the iris-UCA antenna and name our approximation model of a ULA antenna as the iris-ULA antenna.

## 4. Channel Models

We consider that the radio channel is mainly affected by the path loss effect and the shadowing effect [48]. We denote the transmitting power by $P_{t}$ and the receiving power by $P_{r}$. Then, $P_{r}$ can be calculated by:

(15) $$P_{r}=\frac{P_{t}G_{r}G_{t}}{10^{\omega /10}d^{\alpha }},$$

where *d* is the distance between the transmitter and the receiver, *α* is the path loss exponent (usually 2≤α≤6 [49]) and $G_{t}$ and $G_{r}$ are the antenna gains of a transmitter and a receiver, respectively. Besides, $10^{\omega /10}$ is the unit conversion of shadowing effects factor *ω*, which is a Gaussian random variable with zero mean and standard deviation *σ*(dB) (*σ* ranges from 4–12 dB).

In practice, we usually calculate the power attenuation (denoted by *β*) between two nodes instead of computing the received power $P_{r}$. We then derive the power attenuation *β* as follows,

(16) $$\beta =\frac{P_{t}}{P_{r}}=\frac{d^{\alpha }10^{\omega /10}}{G_{t}G_{r}}.$$

The signal can be received successfully if the power attenuation *β* is no greater than a threshold $\beta_{0}$. Therefore, if we substitute *β* in Equation (16) with $\beta_{0}$, we can have the maximum transmission distance $d_{\max}$ as follows,

(17) $$d_{\max}=\;\sqrt[{\alpha }]{\frac{G_{r}G_{t}\beta_{0}}{10^{\omega /10}}}.$$

It is shown in the above analysis that the maximum distance $d_{\max}$ depends on the antenna gains of the transmitter and the receiver. Therefore, $d_{\max}$ varies with the different directions since $G_{r}$ and $G_{t}$ vary in different directions.

## 5. Local Connectivity

In this section, we analyze the local network connectivity of WSNs with various antenna models. Section 5.1 first presents the analytical results on the local network connectivity of WSNs. Section 5.2 then gives the simulation results.

### 5.1. Probability of Isolation

We have the assumption that all of the nodes are distributed according to a homogeneous Poisson point process with density *ρ* in the 2D plane. The number of nodes in an area A is denoted by a random variable *N*. We then have the probability mass function of *N* as follows,

(18) $$f_{N}\left(n\right)=\frac{{\left(\rho A\right)}^{n}}{n!}e^{-\rho A}.$$

We then define the probability of node isolation, which measures the local network connectivity.

**Definition** **2.** *The probability of node isolation* P(iso) *is the probability that for each node in a network, there is no connection to any other node.*

The probability of node isolation is an important metric to evaluate the local network connectivity since it only concerns the probability that a node does not connect with any of its neighbors. It is shown in previous studies [2,19] that a network with a node distribution following a homogeneous Poisson process, P(iso), is given as follows:

(19) $$P\left(\text{iso}\right)=e^{-E[D]},$$

where *D* denotes the node degree, which is defined as the number of nodes that any given nodes can connect directly, and E[·] denotes the statistical expectation. It is obvious that the node degree *D* also follows a Poisson distribution with parameter $\rho E[\pi d_{\max}^{2}]$. Thus, the average node degree can be expressed by:

(20) $$E\left[D\right]=\rho E\left[\pi d_{\max}^{2}\right]=\rho \pi E\left[d_{\max}^{2}\right].$$

After substituting $d_{\max}$ in Equation (20) by the RHS of Equation (17), we have E[D], which can be calculated by the following equation,

(21) $$E\left[D\right]=\rho \pi {\beta_{0}}^{\frac{2}{\alpha }}E\left[10^{-\frac{\omega }{5\alpha }}\right]E\left[{\left(G_{r}G_{t}\right)}^{\frac{2}{\alpha }}\right].$$

It is shown in Equation (21) that the probability of node isolation is mainly affected by two factors: (i) the shadow fading component denoted by $E[10^{-\frac{\omega }{5\alpha }}]$, which depends on both the shadowing effect and the path loss effect; and (ii) the antenna gain component denoted by $E[{\left(G_{r}G_{t}\right)}^{\frac{2}{\alpha }}]$, which depends on the transmitter antenna gain, the receiver antenna gain and the path loss effect. We next investigate the impacts of these two components.

The shadow fading component can be expressed as follows as proven in [19]:

(22) $$E\left[10^{-\frac{\omega }{5\alpha }}\right]=\exp\left\{\frac{{\left(\frac{\ln10}{5\alpha }\sigma \right)}^{2}}{2}\right\}.$$

It is shown in Equation (22) that the shadow fading component depends on both the path loss factor *α* and the log-normal standard deviation *σ*. In addition, we can see that the shadow fading component is always positive, which implies that the shadow fading effect always leads to the increment of the average node degree E[D] (given by Equation (21)).

We next analyze the impacts of the antenna gain component. Like previous studies [19,38], we consider the Randomly-Directed Antenna Scheme (RDAS), in which each node in the network can randomly choose its main beam direction. RDAS can be easily implemented in a distributed network since it requires no pre-knowledge of geographical locations of all nodes.

**Figure 7:**
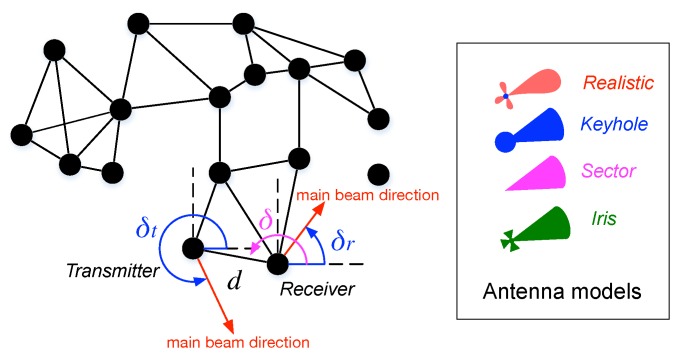
Relative positions of a transmitter and a receiver.

Figure 7 shows the relative positions of a transmitter and a receiver in a network, where *d* is the distance between the transmitter and receiver, *δ* is the angle between the transmitter and the receiver and $\delta_{t}$ and $\delta_{r}$ are the directions of main beams of the transmitter and the receiver, respectively. Note that we denote the main beam directions of the transmitter and the receiver by red arrows as shown in Figure 7. In RDAS, *δ*, $\delta_{t}$ and $\delta_{r}$ are uniformly distributed in (0,2π]. Therefore, the antenna gain effect $E[{\left(G_{r}G_{t}\right)}^{\frac{2}{\alpha }}]$ can be calculated as follows:

(23) $$E\left[{\left(G_{r}G_{t}\right)}^{\frac{2}{\alpha }}\right]=\frac{1}{{\left(2\pi \right)}^{3}}\cdot \int_{0}^{2\pi }\int_{0}^{2\pi }\int_{0}^{2\pi }{\left(G\left(\delta ,\delta_{t}\right)G\left(\pi +\delta ,\delta_{r}\right)\right)}^{\frac{2}{\alpha }}d\delta_{t}d\delta_{r}d\delta ,$$

where $G(\delta ,\delta_{t})$ and $G(\pi +\delta ,\delta_{r})$ are the antenna gains of the transmitter and the receiver, respectively.

Note that Equation (23) can be applied to both realistic antennas (such UCA and ULA antennas) and simplified antenna models (such as keyhole, sector and iris models), as depicted in Figure 7. However, there is no closed-form expression of Equation (23) for realistic antenna models. Table 2 shows the numerical values of $E[{\left(G_{r}G_{t}\right)}^{\frac{2}{\alpha }}]$ of the UCA antenna, keyhole, sector and iris-UCA models; it also gives the deviations of the values of the keyhole, sector and iris-UCA models in contrast to the UCA antenna. These results are obtained by choosing different values of the path loss factor *α* in Equation (23). Note that when the path loss factor is increased to be greater than four, the trend of the antenna gain component becomes less discernible. Thus, we omit the results for α=5 and α=6 here. Table 2 also lists the mean absolute deviation of the effective antenna gain of each antenna.

It is shown in Table 2 that the keyhole model always has higher values of $E[{\left(G_{r}G_{t}\right)}^{\frac{2}{\alpha }}]$ than the UCA antenna, while iris-UCA always has lower values of $E[{\left(G_{r}G_{t}\right)}^{\frac{2}{\alpha }}]$ than the UCA antenna. This implies that keyhole model may overestimate the impacts of antenna gains, and the iris-UCA model may underestimate the impacts of antenna gains. This implication will be confirmed by our simulation results in Section 5.2. On the other hand, the sector model has higher values of $E[{\left(G_{r}G_{t}\right)}^{\frac{2}{\alpha }}]$ than the UCA antenna when *α* is within [2,3.25], and it has lower values of $E[{\left(G_{r}G_{t}\right)}^{\frac{2}{\alpha }}]$ than the UCA antenna when *α* is greater than 3.5. Furthermore, it is shown in Table 2 that our proposed iris-UCA model has the smallest mean absolute deviation of the antenna gain factor from those of the UCA antenna since the mean absolute deviation of iris-UCA is 26.76%, while the mean absolute deviation of the keyhole model is 47.67%, and the mean absolute deviation of sector model is 284.14%. This implies that our proposed iris model has the best approximation of a UCA antenna compared to other existing antenna models.

Table 3 shows different values of $E[{\left(G_{r}G_{t}\right)}^{\frac{2}{\alpha }}]$ of the ULA antenna and iris-ULA antenna and deviations of $E[{\left(G_{r}G_{t}\right)}^{\frac{2}{\alpha }}]$ of the iris-ULA antenna compared with ULA antenna. Note that there is no comparison with keyhole and sector models since they cannot be applied for the ULA antenna. It is shown in Table 3 that the iris-ULA model has a good approximation of a ULA antenna since the mean absolute deviation in contrast to the ULA antenna is 13.39%, which is even lower than that of the iris-UCA model in contrast to the UCA antenna.

### 5.2. Empirical Results of Local Connectivity

In this section, we conduct extensive simulations to verify our analysis and compare the local network connectivity with different antenna models, including UCA and ULA antennas, as well as keyhole, sector, iris-UCA and iris-ULA models. Our simulations are conducted in a MATLAB simulator. In the simulations, nodes are randomly distributed on a plane of area l×l m^2^. To minimize the impacts of the border effect, we use the subarea approach [2], in which we only consider the nodes within an inner square of area $l^{\prime }\times l^{\prime }$ m^2^ ($l^{\prime }$ must be sufficiently smaller than *l*). For example, for the network using the sector model, we consider *l* = 12,000 m, and $l^{\prime }$ = 1000 m. Besides, each value of the probability of node isolation is obtained by averaging over a large number of random topologies (e.g., 5000). Note that we fixed the threshold attenuation $\beta_{0}=50\;\text{dB}$ in all simulations. Table 4 lists the detailed parameters in simulations.

In our simulations, the probability of node isolation is calculated as follows,

(24) $$P_{s}\left(\text{iso}\right)=\frac{\#\;\text{the nodes of isolation}}{\#\;\text{the total nodes}},$$

where # represents “the number of”, and we denote the simulation results of the probability of node isolation by $P_{s}\left(\text{iso}\right)$ in order to differentiate it from the analytical value of P(iso).

#### 5.2.1. Comparisons of the Probability of Node Isolation with UCA Antennas, Keyhole, Sector and Iris-UCA Models

**Figure 8:**
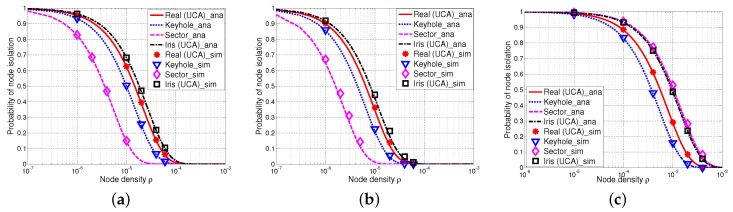
Probability of node isolation P(iso) with UCA antenna, keyhole, sector and iris-UCA models, where curves are analytical results and markers are simulation results. (**a**) α=2.5, σ=4; (**b**) α=2.5, σ=8; (**c**) α=4, σ=8.

Figure 8 shows the probability of node isolation versus the node density with different values of the path loss exponent *α* and the shadow fading factor *σ*, where the analytical results are shown by curves and the simulation results are shown by markers. In particular, it is shown in Figure 8 that the simulation results are in a good agreement with the analytical results in all cases (see Figure 8a–c). Besides, we have found that P(iso) decreases as *σ* increases with a fixed *α* (e.g., Figure 8a,b). This is because the shadow fading effect leads to the increment of the shadow fading component, consequently decreasing the probability of node isolation. This trend further confirms our previous observations in Section 5.1. Moreover, P(iso) significantly increases with the increment of the path loss effect *α*, which also matches the previous findings in [19,38].

Figure 8 also shows that keyhole, sector and our proposed iris-UCA models perform differently in terms of P(iso). In particular, the values of P(iso) of the keyhole model are always lower than those of realistic UCA antenna, while the results of our proposed iris-UCA model are always higher than those of the realistic UCA antenna. However, our iris-UCA model has the smallest average deviation from realistic UCA antenna among all of the antenna models, especially when the path loss is less significant (e.g., α≤3). Besides, the results of the sector model always have the highest deviations from those of the realistic UCA antenna compared with the keyhole and iris-UCA models.

In summary, our iris-UCA model provides a relatively better approximation to the realistic UCA antenna compared with keyhole and sector models on average. These results are also consistent with the analysis of antenna components $E[{\left(G_{r}G_{t}\right)}^{\frac{2}{\alpha }}]$ (as shown in Table 2). Among the three simplified antenna models (keyhole, sector and iris-UCA models), our proposed iris-UCA model has the best approximation to UCA antenna on average in terms of P(iso). These observations are also consistent with our earlier analysis in Section 5.1.

#### 5.2.2. Comparisons of the Probability of Node Isolation with the ULA Antenna and Iris-ULA Model

**Figure 9:**
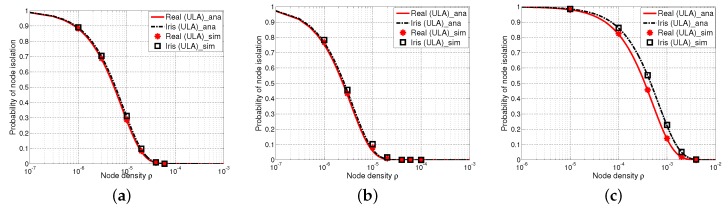
Probability of node isolation P(iso) with the ULA antenna and iris-ULA model, where curves are analytical results and markers are simulation results. (**a**) α=2.5, σ=4; (**b**) α=2.5, σ=8; (**c**) α=4, σ=8.

Figure 9 shows the probability of node isolation versus the node density with different values of the path loss exponent *α* and the shadow fading factor *σ* with the ULA antenna and our proposed iris-ULA model. We can see that the simulation results and analytical results are also in a good agreement, implying that our iris-ULA model is also quite accurate.

It is shown in Figure 9 that the probability of node isolation of the iris-ULA model has the best approximation of that of the ULA antenna. For example, the results of the iris-ULA model are just slightly higher than those of the ULA antenna when α=2.5 and σ=4, as shown in Figure 9a, and when α=2.5 and σ=8, as shown in Figure 9b. When *α* is increased to be more than four, the deviation of the results of the iris-ULA model from those of ULA antenna is further expanded, as shown in Figure 9c. These results are in a good agreement with our previous analysis of antenna gain components $E[{\left(G_{r}G_{t}\right)}^{\frac{2}{\alpha }}]$ in Section 5.1.

## 6. Overall Connectivity

In this section, we investigate the network connectivity from a global point of view of all nodes. In particular, we derive the analytical results of one-connectivity in Section 6.1 and present the simulation results in Section 6.2.

### 6.1. One-Connectivity

We measure the overall network connectivity by one-connectivity, which is formally defined as follows.

**Definition** **3.** *One-connectivity* P(1-con) *is the probability that for each node pair, there exists at least one path connecting them.*

As shown in [2], one-connectivity is a special case of *k*-connectivity (i.e., in a *k*-connected network, each node pair has at least *k* node-disjoint paths connecting them). We then derive the relation between one-connectivity and the probability of node isolation. In particular, we have that the non-existence of isolated nodes is a necessary condition, but not a sufficient condition for a network to be connected. Thus, the probability of no isolated nodes in a network, denoted by P(no node isolation), is the upper bound of P(1-con), as shown in [2,30],

(25) $$\begin{array}{c} \rho (P(1-\text{con})=p)=\rho (P(\text{no node isolation})=p)+\epsilon \\ \text{with}\;\epsilon \geq 0\;\text{and}\;\epsilon \to 0\;\text{as}\;p\to 1, \end{array}$$

where *ρ* is the node density.

On the other hand, P(no node isolation) can be expressed as:

(26) P(no node isolation)=exp{−ρAP(iso)},

where A is the area of the network and P(iso) is the probability of node isolation, which is defined in Equation (19) in Section 5.1.

As indicated in Equations (25) and (26), there is a strong connection between the probability of node isolation P(iso) and P(1-con), implying that the overall network connectivity heavily depends on the local network connectivity.

As shown in [2,30], it is sufficient to compute the minimum node density *ρ*, such that P(no node isolation) = 99%, and we can use this node density as a tight bound for the node density, such that P(1-con) = 99%. The minimum node density is called the critical node density denoted by $\rho_{c}$. The critical node density $\rho_{c}$ can be solved from Equation (26) as follows:

$$\begin{array}{cc} \rho_{c}= & -\frac{1}{E[\pi d_{\max}^{2}]}W_{-1}\left(\frac{E[\pi d_{\max}^{2}]\ln0.99}{A}\right)\\ = & -\frac{1}{\pi {\delta_{0}}^{\frac{2}{\alpha }}E\left[10^{-\frac{\omega }{5\alpha }}\right]E\left[{\left(G_{r}G_{t}\right)}^{\frac{2}{\alpha }}\right]}\cdot W_{-1}\left(\frac{\ln0.99}{A}\pi {\delta_{0}}^{\frac{2}{\alpha }}E\left[10^{-\frac{\omega }{5\alpha }}\right]E\left[{\left(G_{r}G_{t}\right)}^{\frac{2}{\alpha }}\right]\right), \end{array}$$

where $W_{-1}$ denotes the real-valued non-principal branch of Lambert’s W function [50].

The higher $\rho_{c}$ implies that the network needs more nodes to keep the network connected, i.e., each node can be connected with each other. In other words, the higher $\rho_{c}$ implies the lower overall network connectivity.

Table 5 and Table 6 present the results on the critical node density $\rho_{c}$ with the shadowing factor σ=4 and σ=8, respectively. We also give the deviations of keyhole, sector and iris-UCA models in contrast to the realistic UCA antenna. The deviations are evaluated in percentage compared with the values of UCA antenna.

As shown in Table 5 and Table 6, we can see that the critical node density $\rho_{c}$ varies with different antenna models, different values of the path loss exponent *α* and the shadow fading factor *σ*. This implies that the overall network connectivity heavily depends on various factors, such as the antenna models and the channel randomness (such as the path loss effect and the shadow fading effect). In particular, we find that the higher the path loss exponent *α* is, the higher the critical node density $\rho_{c}$ is required to ensure the network connectivity, implying that the higher path loss effect results in the lower network connectivity. On the contrary, the critical node density decreases with the increment of shadow fading factor *σ* (see Table 5 and Table 6) if we fix the other factors, implying that the higher shadow fading variance leads to the higher network connectivity, which confirms the previous results in [30].

Next, let us have a look at the impacts of different antenna models on the critical node density. In particular, as shown in Table 5 and Table 6, the keyhole model always has negative deviations in the critical node density $\rho_{c}$ compared with the realistic UCA antenna, implying that the keyhole model results in higher network connectivity. On the contrary, the iris model always has positive deviations in the critical node density $\rho_{c}$ compared with the realistic UCA antenna, implying that it will lead to the lower network connectivity. Different from keyhole and iris models, the sector model has negative deviations when α≤3 and has positive deviations when α>3. Besides, the trend of the deviations of $\rho_{c}$ is less susceptible to the shadow fading variance *σ* when we compare Table 5 with Table 6. Furthermore, we also find that the keyhole model has relatively stable deviations of $\rho_{c}$ compared with sector and iris models (e.g., the deviations of keyhole are about 35%). Moreover, the iris model has the lowest deviations when the path loss effect is not that notable (e.g., α≤2.5).

Table 7 and Table 8 present the results on the critical node density $\rho_{c}$ with the realistic ULA antenna and the iris-ULA model when the shadowing factor σ=4 and σ=8, respectively. Note that keyhole and sector models cannot be used to approximate the ULA antenna. Similarly, Table 7 and Table 8 also give the deviations of iris-ULA in contrast to realistic ULA antenna, where we have similar findings to the UCA results. For example, the critical node density always increases with the increased path loss effect (the increment of the path loss exponent *α*). Slightly different from the iris-UCA model, the iris-ULA model has a negative deviation when α=2 and positive deviations when α≥2.5. Besides, the iris-ULA model reduces the deviations compared with the iris-UCA model. For example, the maximum deviation in the iris-ULA model is +35.97% in contrast to +81.23% in the iris-UCA model when σ=4. This implies that the iris model may offer a better approximation to the ULA antenna than that to the UCA antenna.

### 6.2. Empirical Results of One-Connectivity

In this section, we conduct extensive simulations in MATLAB to verify our analysis and compare the one-connectivity with different antenna models, including UCA and ULA antennas, as well as keyhole, sector, iris-UCA and iris-ULA models. The detailed parameters of simulations are presented in Table 9.

To differentiate it from the analytical value of P(1-con), we denote the one-connectivity of the simulation results by $P_{s}$(1-con), which can be calculated by the following equation,

(27) $$P_{s}\left(1-\text{con}\right)=\frac{\text{the number of connected topologies}}{\text{the total number of random topologies}}.$$

It is shown in [2] that Equation (27) may have a good estimation of P(1-con) for a sufficiently large number of random topologies.

#### 6.2.1. Comparisons of One-Connectivity with UCA Antenna, Keyhole, Sector and Iris-UCA Models

**Figure 10:**
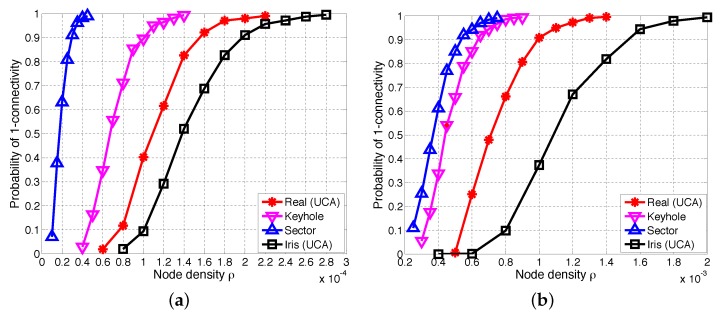
One-connectivity P(1-con) of UCA antenna, keyhole, sector and iris-UCA models. (**a**) α=2.5, σ=4; (**b**) α=3, σ=4.

Figure 10 presents the simulation results of the one-connectivity of UCA antenna, keyhole, sector and iris-UCA models with different values of the path loss exponent *α* and the shadow fading variance *σ*. In particular, both keyhole and sector models have curves of P(1-con) above those of UCA antennas, while the iris-UCA model has curves below those of UCA antennas. Besides, the sector model always has much higher P(1-con) than those of other simplified models, implying that the sector model may be less accurate than other models. These results agree with our earlier expectations in Section 6.1 (see Table 5 and Table 6). Moreover, Figure 10 also indicates that compared with keyhole and sector models, the iris-UCA model has a better approximation of UCA antenna when the path loss effect is small (i.e., α=2.5), while this advantage of the iris-UCA model is not that notable when the path loss effect is further increased to α=3.

#### 6.2.2. Comparisons of One-Connectivity with ULA Antenna and Iris-ULA Model

**Figure 11:**
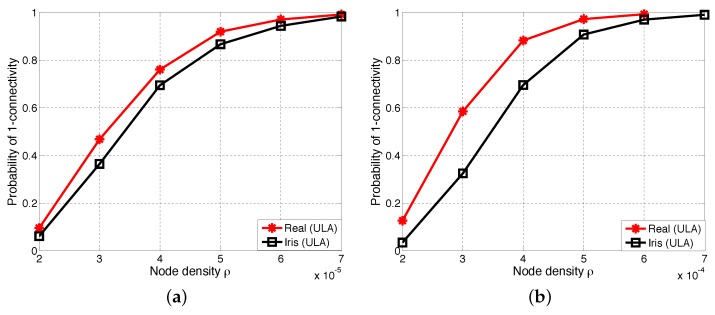
One-connectivity P(1-con) of the ULA antenna and iris-ULA model. (**a**) α=2.5, σ=4; (**b**) α=3, σ=4.

We conduct simulations to evaluate the one-connectivity with the comparison of the ULA antenna and iris-ULA model. Figure 11 presents the simulation results with different values of the path loss exponent *α* and the shadow fading variance *σ*. As shown in Figure 11, we can see that the P(1-con) curves of the ULA antenna are always above those of the iris-ULA model. This trend also agrees with our previous observations in Section 6.1 (see Table 7 and Table 8). Besides, when the path loss exponent *α* increases from 2.5–3, we can see that the gap between the curves of ULA antenna and the curves of iris-ULA is further widened.

## 7. Discussion and Future Directions

Conventional WSNs now have many new applications, among which wireless body sensor networks (WBSNs) are one of the most promising technologies. In WBSNs, sensors usually communicate with a local hub in the single-hop manner [51,52,53]. However, it is shown in [54,55] that in the next generation of WBSNs, some sensors can serve as relays to communicate with each other in the multi-hop fashion with collaboration. These multi-hop WBSNs can be extended from conventional health care services (i.e., e-health) to other emerging applications, such as emergency services, entertainment and military use [55], which are featured with the better flexibility and location independence.

How to improve the connectivity and to enhance the energy efficiency are essential for WBSNs. Using directional antennas in multi-hop WBSNs can potentially improve the performance of WBSNs. For example, it is shown in [56] that using the beamforming scheme can improve the energy efficiency of WBSNs. However, to the best of our knowledge, there are few studies on investigating the connectivity of WBSNs with directional antennas. Hence, our study on investigating the connectivity of WSNs with various directional antenna models can potentially bridge this technical gap.

## 8. Conclusions

In this paper, we investigate the network connectivity of WSNs with different antenna models under the channel with the consideration of the path loss effect and the shadow fading effect. In particular, we propose the iris model, which can approximate almost any type of directional antenna, since there is no restriction on the number of main lobes and side lobes in our iris model. More specifically, we consider both the local network connectivity and the overall network connectivity to evaluate the impacts of different antenna models. Our extensive simulations show that the analytical framework can accurately model both the local connectivity and the overall connectivity. Besides, our results also show that on average, our proposed iris antenna model offers a better approximation to realistic directional antennas (e.g., UCA antennas and ULA antennas) than other existing simplified antenna models, especially when the path loss effect is not significant (i.e., the path loss exponent *α* is smaller than three).

## Figures and Tables

**Table 1:** Summary of simplified directional antenna models.

Features	Keyhole Model	Sector Model	Iris Model (This Paper)
Main beam	Yes	Yes	**Yes**
Side/back lobes	Yes	No	**Yes**
Nulling capability	No	Yes	**Yes**
More than one main beam	No	No	**Yes**

**Table 2:** $E[{\left(G_{r}G_{t}\right)}^{\frac{2}{\alpha }}]$ of the UCA antenna, keyhole, sector and iris-UCA with deviations compared with the UCA antenna.

Path Loss *α*	Antenna Models			
	UCA	Keyhole	Sector	Iris-UCA
2	1.61	2.33 (+44.34%)	22.60 (+1302.28%)	1.55 (−3.67%)
2.25	1.32	1.92 (+45.31%)	9.42 (+612.86%)	1.16 (−12.28%)
2.5	1.15	1.68 (+46.59%)	4.68 (+307.40%)	0.93 (−19.15%)
2.75	1.04	1.53 (+47.72%)	2.64 (+154.39%)	0.78 (−24.74%)
3	0.96	1.43 (+48.55%)	1.64 (+70.14%)	0.68 (−29.39%)
3.25	0.91	1.36 (+49.06%)	1.09 (+20.16%)	0.61 (−33.31%)
3.5	0.87	1.30 (+49.28%)	0.77 (−11.33%)	0.55 (−36.67%)
3.75	0.84	1.26 (+49.23%)	0.57 (−32.18%)	0.51 (−39.56%)
4	0.82	1.23 (+48.98%)	0.44 (−46.54%)	0.48 (−42.08%)
Mean absolute deviation	N/A	47.67%	284.14%	26.76%

**Table 3:** $E[{\left(G_{r}G_{t}\right)}^{\frac{2}{\alpha }}]$ of the ULA antenna and iris-ULA antenna with deviations compared with the ULA antenna.

Path Loss *α*	Antenna Models	
	Realistic ULA	Iris-ULA
2	6.07	6.26 (+3.23%)
2.25	4.13	4.04 (−2.10%)
2.5	3.08	2.87 (−6.64%)
2.75	2.44	2.19 (−10.58%)
3	2.04	1.75 (−14.04%)
3.25	1.76	1.46 (−17.12%)
3.5	1.56	1.25 (−19.87%)
3.75	1.42	1.10 (−22.36%)
4	1.31	0.99 (−24.61%)
Mean absolute deviation	N/A	13.39%

**Table 4:** Parameters in simulations of local connectivity.

Parameters	Values
Number of topologies	5000
Attenuation threshold $\beta_{0}$	50 dB
Path loss exponent *α*	2.5, 4
Standard deviation of shadow effect *σ*	4, 8

**Table 5:** Critical node density $\rho_{c}$ with UCA antenna, keyhole, sector and iris-UCA models when σ=4.

*α*	*A* (m^2^)	Antenna Models			
		Realistic UCA	Keyhole	Sector	Iris-UCA
2	10^6^	8.75 × 10^−6^	5.67 × 10^−6^ (−35.20%)	3.18 × 10^−7^ (−96.37%)	9.14 × 10^−6^ (+4.46%)
2.5	10^6^	2.10 × 10^−4^	1.37 × 10^−4^ (−34.76%)	4.34 × 10^−5^ (−79.33%)	2.66 × 10^−4^ (+26.67%)
3	2.5 × 10^5^	1.32 × 10^−3^	8.58 × 10^−4^ (−35.00%)	7.38 × 10^−4^ (−44.09%)	1.90 × 10^−3^ (+43.94%)
3.5	2.5 × 10^5^	5.20 × 10^−3^	3.35 × 10^−3^ (−35.50%)	5.93 × 10^−3^ (+14.04%)	8.56 × 10^−4^ (+64.57%)
4	2.5 × 10^5^	1.40 × 10−2	9.10 × 10^−3^ (−35.15%)	2.76 × 10−2 (+97.01%)	2.54 × 10−2 (+80.67%)

**Table 6:** Critical node density $\rho_{c}$ with UCA antenna, keyhole, sector and iris-UCA models when σ=8.

*α*	A (m^2^)	Antenna Models			
		Realistic UCA	Keyhole	Sector	Iris-UCA
2	10^6^	1.90 × 10^−6^	1.20 × 10^−5^ (−36.84%)	2.48 × 10^−8^ (−98.69%)	1.99 × 10^−6^ (+4.74%)
2.5	10^6^	8.46 × 10^−5^	5.50 × 10^−5^ (−34.99%)	1.71 × 10^−5^ (−79.79%)	1.07 × 10^−4^ (+26.48%)
3	2.5 × 10^5^	7.06 × 10^−4^	4.57 × 10^−4^ (−35.27%)	3.92 × 10^−4^ (−44.48%)	1.00 × 10^−3^ (+41.64%)
3.5	2.5 × 10^5^	3.30 × 10^−3^	2.12 × 10^−3^ (−35.61%)	3.77 × 10^−3^ (+14.10%)	5.44 × 10^−3^ (+64.86%)
4	2.5 × 10^5^	9.93 × 10^−3^	6.43 × 10^−3^ (−35.22%)	1.96 × 10^−2^ (+97.31%)	1.80 × 10^−2^ (+81.23%)

**Table 7:** Critical node density $\rho_{c}$ with the ULA antenna and the iris-ULA model when σ=4.

*α*	A (m^2^)	Antenna Models	
		Realistic ULA	Iris-ULA Model
2	10^6^	1.78 × 10^−6^	1.71 × 10^−6^ (−3.86%)
2.5	10^6^	6.98 × 10^−5^	7.54 × 10^−5^ (+8.05%)
3	2.5 × 10^5^	5.75 × 10^−4^	6.81 × 10^−4^ (+18.39%)
3.5	2.5 × 10^5^	2.74 × 10^−3^	3.50 × 10^−3^ (+27.53%)
4	2.5 × 10^5^	8.48 × 10^−3^	1.15 × 10^−3^ (+35.97%)

**Table 8:** Critical node density $\rho_{c}$ with the ULA antenna and the iris-ULA model when σ=8.

*α*	A (m^2^)	Antenna Models	
		Realistic ULA	Iris-ULA
2	10^6^	3.38 × 10^−6^	3.23 × 10^−6^ (−4.44%)
2.5	10^6^	2.77 × 10^−5^	2.99 × 10^−5^ (+7.94%)
3	2.5 × 10^5^	3.05 × 10^−4^	3.62 × 10^−4^ (+18.69%)
3.5	2.5 × 10^5^	1.70 × 10^−3^	2.70 × 10^−3^ (+29.41%)
4	2.5 × 10^5^	6.00 × 10^−3^	8.20 × 10^−3^ (+36.67%)

**Table 9:** Parameters in simulations of one-connectivity.

Parameters	Values
Number of topologies	5000
Attenuation threshold $\beta_{0}$	50 dB
Path loss exponent *α*	2.5, 3
Standard deviation of shadow effect *σ*	4
